# Apoptosis of Hepatocellular Carcinoma Cells Induced by Nanoencapsulated Polysaccharides Extracted from *Antrodia Camphorata*

**DOI:** 10.1371/journal.pone.0136782

**Published:** 2015-09-01

**Authors:** Jenq-Sheng Chang, Hsiang-Ping Kuo, Ke Liang B. Chang, Zwe-Ling Kong

**Affiliations:** Department of Food Science, National Taiwan Ocean University, Keelung, Taiwan; University of Windsor, CANADA

## Abstract

*Antrodia camphorata* is a well-known medicinal mushroom in Taiwan and has been studied for decades, especially with focus on anti-cancer activity. Polysaccharides are the major bioactive compounds reported with anti-cancer activity, but the debates on how they target cells still remain. Research addressing the encapsulation of polysaccharides from *A*. *camphorata* extract (ACE) to enhance anti-cancer activity is rare. In this study, ACE polysaccharides were nano-encapsulated in chitosan-silica and silica (expressed as ACE/CS and ACE/S, respectively) to evaluate the apoptosis effect on a hepatoma cell line (Hep G2). The results showed that ACE polysaccharides, ACE/CS and ACE/S all could damage the Hep G2 cell membrane and cause cell death, especially in the ACE/CS group. In apoptosis assays, DNA fragmentation and sub-G_1_ phase populations were increased, and the mitochondrial membrane potential decreased significantly after treatments. ACE/CS and ACE/S could also increase reactive oxygen species (ROS) generation, induce Fas/APO-1 (apoptosis antigen 1) expression and elevate the proteolytic activities of caspase-3, caspase-8 and caspase-9 in Hep G2 cells. Unsurprisingly, ACE/CS induced a similar apoptosis mechanism at a lower dosage (ACE polysaccharides = 13.2 μg/mL) than those of ACE/S (ACE polysaccharides = 21.2 μg/mL) and ACE polysaccharides (25 μg/mL). Therefore, the encapsulation of ACE polysaccharides by chitosan-silica nanoparticles may provide a viable approach for enhancing anti-tumor efficacy in liver cancer cells.

## Introduction

Medicinal mushrooms have become increasingly popular in recent years for their potential in disease prevention, especially in tumor inhibition [1, 2]. *Antrodia camphorata* is a well-known mushroom that has been used as an herbal medicine for centuries in Taiwan. This mushroom has been used in the treatment of various diseases; e.g., diarrhea, abdominal pain and hypertension [3, 4]. Moreover, the pharmacological properties of *A*. *camphorata*, such as hepatoprotective properties [5, 6] and anti-tumor activities [7–9], have been stated and summarized. Generally, polysaccharides and triterpenoids are the major bioactive components in medicinal mushrooms, and the polysaccharides extracted from *A*. *camphorata* were found to have anti-hepatitis B surface antigen [10] and anti-tumor effects [3, 11].

Rapid development in nanotechnology engenders a variety of nanoparticles with novel bioactive ingredients delivered into cancer cells [12]. Natural and synthetic polymers as well as inorganic materials could be used for constructing functional nanoparticles. Among these materials, silica and chitosan have received more attention because of their abundance in nature. Chitosan is a cationic polysaccharide with attractive chemical and biological characteristics for pharmaceutical purposes [13] and has been incorporated into various carrier systems for drug delivery to improve their absorption and targeting characteristics [14–20].

Silica nanostructures allow one to encapsulate biomolecules and therefore deliver multiple clinical functions. Most researchers synthesize silica with various sizes, morphology and surface functional properties of silicon alkoxide by the sol-gel process [21, 22]. In the past decade, a variety of silicate-based biohybrid materials containing silica and biopolymers have been produced and are widely used in bio-encapsulation applications due to the low environmental impact, low toxicity and good biocompatibility [23]. We recently demonstrated that chitosan was capable of forming composite nanoparticles with silica [24]. Moreover, the presence of chitosan in the composite, i.e., chitosan-silica nanoparticles, significantly reduced the cytotoxicity of silica nanoparticles [25]. Nevertheless, little information is currently available on changes in the efficacy of bioactive ingredients encapsulated by biopolymer-silica hybrid nanoparticles.

The aim of this study was to evaluate the effects of *A*. *camphorata* extract (ACE) polysaccharides, ACE polysaccharides encapsulated in chitosan-silica nanoparticles (ACE/CS) and encapsulated in silica nanoparticles (ACE/S) on apoptosis of cells in a human liver cancer cell line (Hep G2). We investigated the cell cycle, mitochondrial membrane potential, Fas/APO-1, caspase-8, caspase-9 and caspase-3 signaling molecules, which are strongly associated with the apoptosis signal transduction pathway and are related to the responses of tumor cells treated with anti-cancer compounds.

## Materials and Methods

### Reagents and chemicals

The chitosan samples were purchased from Lytone Enterprise Inc. (Taipei, Taiwan). Sodium acetate, acetic acid, alcohol and potassium bromide were all from MERCK (Darmstadt, Germany). Sodium silicate solution (Si = 52%–57%) and hexamine were obtained from Wako Chemical (Osaka, Japan). Sodium phosphate, 3-(4, 5-dimethylthiazol-2-yl)-2, 5-diphenyltetrazolium bromide (MTT) and dichlorofluorescin diacetate (DCFDA) were purchased from Sigma (St. Louis, MO, USA). The CytoTox96 nonradioactive assay kit (LDH assay) was purchased from Promega (Madison, WI, USA). Dulbecco’s modified eagle’s medium (DMEM), Trypsin-EDTA were from Gibco (Carlsbad, California, USA). EXCELL-610-HSF medium was obtained from JRH (KS, USA). Fetal bovine serum was purchased from PAA (Pasching, Austria). Dulbecco’s phosphate buffered saline was from Nissui Pharmaceutical CO., LTD (Taito-ku, Tokyo, Japan). Trail was purchased from PeproTech, Inc. (NJ, USA). Phycoerythin (PE)-conjugated Fas monoclonal antibody UB2 was purchased from LSBio (Seattle WA, USA). CaspGLOW Fluorescein Active Caspase-3 (DEVD-pNA), Caspase-8 (IETD-pNA) and Caspase-9 (LEHD-pNA) Staining Kits were purchased from BioVision (CA, USA). Finally, the Hep G2 (hepatocellular carcinoma) cell line was acquired from the American Type Culture Collection (ATCC).

### Preparations of ACE polysaccharides, ACE/CS and ACE/S

Powdered fruiting bodies of cultivated *A*. *camphorata* (approximately 70 g) were soaked in 500 mL ethanol (70%) for 12 h. The suspension was centrifuged at 5,520× *g* for 30 min to remove the insoluble matter, and the supernatant was concentrated with a vacuum evaporator until the volume reached 100 mL. The above solution was dialyzed (10 KDa MWCO) overnight, filtered with a filter paper (pore size 0.45 μm) and freeze-dried to powder form for further analysis. For nanoparticle preparation, the qualified chitosan samples purchased from a commercial supplier were analyzed for the degree of deacetylation (DD) up to 81% and molecular weight (Mw), which was approximately 200 kDA. ACE/CS was prepared as previously described [24]. Briefly, the sodium silicate was dissolved in 30 mL buffer (0.05 M sodium acetate solution) to prepare a 0.55% (w/w) solution (pH = 6.0). After 10 min agitation in a magnetic stirrer, 6 mL ACE polysaccharides solution (0.1% w/w) and 3 mL chitosan solution (0.55% w/w) were added. The resulting solution was mixed completely and set aside for particle synthesis (4 h) without disturbance. ACE/CS was collected by centrifugation at 5,520× *g* for 30 min. For ACE/S preparation, silica solution at the same concentration described above without chitosan solution was used and ACE/S was collected after 12 h of the particle synthesis process.

### Cell culture

Hep G2 (hepatocellular carcinoma) cells were cultured and maintained in Dulbecco’s modified eagle’s medium (DMEM) with 10% fetal bovine serum (FBS) at 37°C in a humidified 95% air containing 5% CO_2_.

### Cell viability analysis

Hep G2 cells were seeded in 96-well plates at a density of 2.0 × 10^5^ cells/well. After incubation for 8 h, the medium was removed and replaced by 20 μL of different concentrations of ACE polysaccharides, ACE/ES, ACE/S and nanoparticles without loading, which were suspended in deionized water and ultrasonicated for 1 h to prevent agglomeration. Controls were cells treated with an equivalent volume of serum-free medium instead of suspensions. Cells were further incubated for 24 h or 48 h and viability was assessed by using an MTT (3-(4, 5-dimethylthiazol-2-yl)-2, 5-di-phenyltetrazolium bromide) test [26]. In brief, fresh media with 100 μL of 5 mg/mL MTT stain (Sigma-Aldrich in vitro toxicology assay kit) was added into each well and incubated for 4 h. The media/stain was drawn out and purple color crystals were dissolved using acidic isopropyl alcohol. The absorbance in each well was measured at 570 nm using a 4294B Microplate Reader (Tecan Sunrise, American Instrument Exchange Inc. USA). All experiments were repeated 3 times independently to ensure reproducibility and data were acquired in triplicate (n = 3). Cell viability was calculated with the following formula:

$$A570_{sample}/A570_{control}\times 100\%,$$

Where control stands for culture without any treatment.

### Lactate dehydrogenase release assay

Cells at a density of 2.0 × 10^5^ cells/well were treated in the same protocol described above except at the indicated concentrations of suspensions. After 48 h incubation, the cultured cells were centrifuged at 430× *g* for 5 min and the cell medium was transferred to a new 96-well plate (50 μL/well). Upon the addition of the lactate dehydrogenase (LDH) reaction mixture (Promega, CytoTox 96R non-radioactive assay kit) to the wells (50 μL/well), they were kept in the dark for 30 min and then 1 N HCl (25 μL/well) was added to each sample to terminate the reaction. The resulting absorbance was measured at 490 nm. All experiments were repeated 3 times independently to ensure reproducibility and data were acquired in triplicate (n = 3). Control and positive control experiments were performed with medium only and with 0.1% (w/v) Triton X-100, with cytotoxicity identified at 100%, respectively. LDH release (%) was calculated using following equation:

$$\text{LDH }\left(\%\right)=\left[\left(A490_{sample}-A490_{medium}\right)/\left(A490_{100\%}-A490_{medium}\right)\right]\times 100\%$$

### Gel electrophoresis examination for DNA fragments

Hep G2 cells were seeded at the density of 2.0 × 10^5^ cells/mL in 10-cm dishes containing medium supplemented with 2% FBS and incubated for 4 h. Tumor necrosis factor-related apoptosis-inducing ligand (Trail), for positive control, ACE polysaccharides, ACE/CS and ACE/S suspensions were added to the dishes, protected from light and incubated for 48 h. Cells were harvested by centrifugation at 250× *g* after incubation, lysed in buffer solution and centrifuged at 14,000× *g* for 10 min. The fragmented DNAs in the supernatant were extracted and analyzed with 2% agarose gel electrophoresis containing 0.1 μg/mL ethidium bromide. The stained DNA fragments were imaged with Imagemaster VDS (Pharmacia, Uppsala, Sweden).

### Cell cycle

Hep G2 cells were seeded at the density of 1.0 × 10^6^ cells per 60-mm dish and treated with ACE polysaccharides (25 μg/mL), ACE/CS (ACE polysaccharides = 13.2 μg/mL) and ACE/S (ACE polysaccharides = 21.2 μg/mL) for 24 h and 48 h, and then were harvested and fixed in ice cold 70% ethanol (v/v). The cells were further washed with phosphate-buffered saline (pH 7.2), incubated with 25 μg/mL RNase A at 37°C for 15 min and stained with 50 μg/mL propidium iodide (PI) for 30 min in the dark. In the flow cytometry assay, a FACSCalibur Flow Cytometer (Becton Dickinson, BD Biosciences, CA, USA) with an excitation wavelength 488 nm and an emission wavelength 630 nm was used, and data were acquired using Cell Quest software based on a minimum of 10^5^ cells per sample.

### Mitochondrial membrane potential analysis

Again, the Hep G2 cells were treated with ACE polysaccharides (25 μg/mL), ACE/CS (ACE polysaccharides = 13.2 μg/mL) and ACE/S (ACE polysaccharides = 21.2 μg/mL) at 37°C for 48 h. After being resuspended at a density of 1.0 × 10^6^ cells/mL in PBS, the cells were stained with 25 μM Rhodamine 123 for 30 min at 37°C and the membrane potential (ΔΨ) was detected by a FACSCalibur Flow Cytometer with excitation at 488 nm and emission at 520 nm based on a minimum of 10^5^ cells per sample.

### Measurement of intracellular reactive oxygen species

Formation of reactive oxygen species (ROS) in cells was evaluated according to the modified method adapted from Chou and Lin [27]. Briefly, Hep G2 cells were suspended in DMEM/10% FBS with or without ACE polysaccharides (25 μg/mL), ACE/CS (ACE polysaccharides = 13.2 μg/mL) and ACE/S (ACE polysaccharides = 21.2 μg/mL) at 37°C for 48 h. After trypsinization, the cells were washed, resuspended at a density of 1.0 × 10^6^ cells/mL in PBS and then kept in a dark chamber containing DCFDA (20 μM) for further flow cytometric analysis (488 nm excitation/520 nm emission).

### Assay for Fas (apoptosis antigen 1, APO-1/cluster of differentiation 95, CD 95)

Hep G2 cells were treated with or without ACE polysaccharides, ACE/CS and ACE/S in the same concentrations described above at 37°C for 48 h, trypsinized, washed, resuspended at a density of 1.0 × 10^6^ cells/mL in PBS and incubated with phycoerythin (PE)-conjugated Fas monoclonal antibody UB2 (1 pg/mL) at 4°C for 1 h. Samples were then valuated by a FACSCalibur Flow Cytometer with excitation 488 nm/emission 580 nm and data were acquired using Cell Quest software based on a minimum of 10^5^ cells per sample.

### Activity of caspase-3, caspase-8 and caspase-9

Activities of caspase-3, caspase-8 and caspase-9 were determined with a CaspGLOW Fluorescein Active Caspase Staining Kit (BioVision, USA). In brief, cells were pre-incubated with ACE polysaccharides (25 μg/mL), ACE/CS (ACE polysaccharides = 13.2 μg/mL) and ACE/S (ACE polysaccharides = 21.2 μg/mL) for 48 h and counted to 1.0 × 10^6^ cells/60-mm dish. After centrifuging at 400× *g* for 10 min, the cell pellets were lysed and kept on ice for 10 min. Supernatants were collected after centrifuging at 10,000× *g* at 4°C for 3 min and DEVD-pNA (Asp-Glu-Val-Asp p-nitroaniline, for caspase-3), IETD-pNA (Ile-Glu-Thr-Asp p-nitroaniline, for caspase-8), LEHD-pNA (Leu-Glu-His-Asp p-nitroaniline, for caspase-9) being added to a final concentration of 50 mM. Each sample was further incubated at 37°C for 1 h in a water bath and fluorescence was detected by a FACSCalibur Flow Cytometer with excitation at 485 nm and emission at 520 nm based on a minimum of 10^5^ cells per sample.

### Statistical analysis

All data from the experiments were expressed as the mean ± SD and shown with error bars. A one-way ANOVA followed by Duncan’s test was used for significance testing, using a *p* value of 0.05 (SPSS 11, SPSS Inc., Chicago, IL).

## Results

### ACE polysaccharides, ACE/CS and ACE/S induce Hep G2 death and LDH release

**Fig 1:**
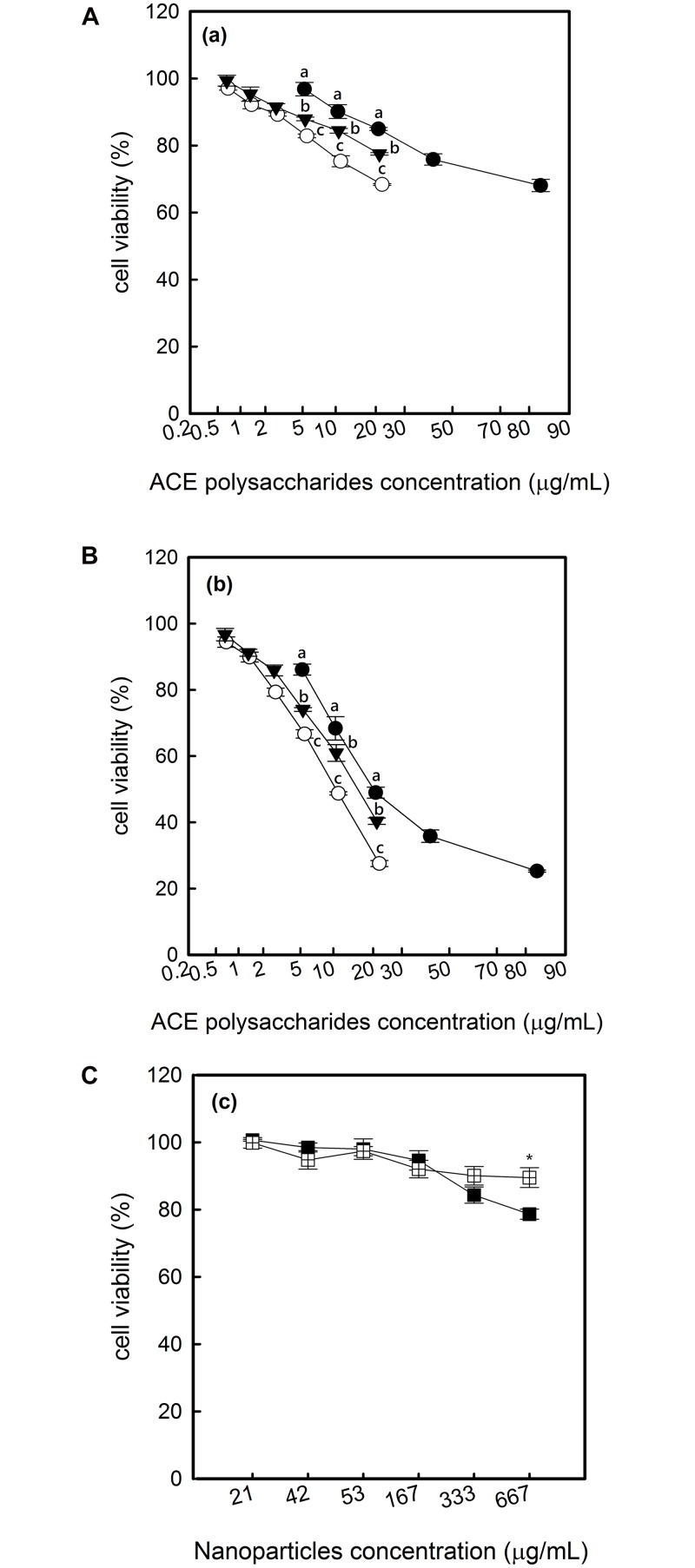
The cytotoxicity of ACE polysaccharides, ACE/CS and ACE/S on Hep G2 cells. Cells were incubated with samples for (a) 24 h or (b) 48 h and viability was assessed using an MTT assay. Nanoparticles without AEC polysaccharides were presented as (c) for 48 h incubation. Experiments were repeated 3 times independently to ensure reproducibility and the standard deviation of the mean are represented as error bars (n = 3). Values with different letters or asterisks were significantly different (*p* < 0.05) at corresponding concentrations between different treatments (●) ACE polysaccharides: *A*. *camphorata* extract, polysaccharides; (○) ACE/CS: ACE polysaccharides encapsulated by chitosan-silica nanoparticles; (▼) ACE/S: ACE polysaccharides encapsulated by silica nanoparticles; (■) SNP: silica nanoparticles; (□) CSNP: chitosan-silica nanoparticles.

**Fig 2:**
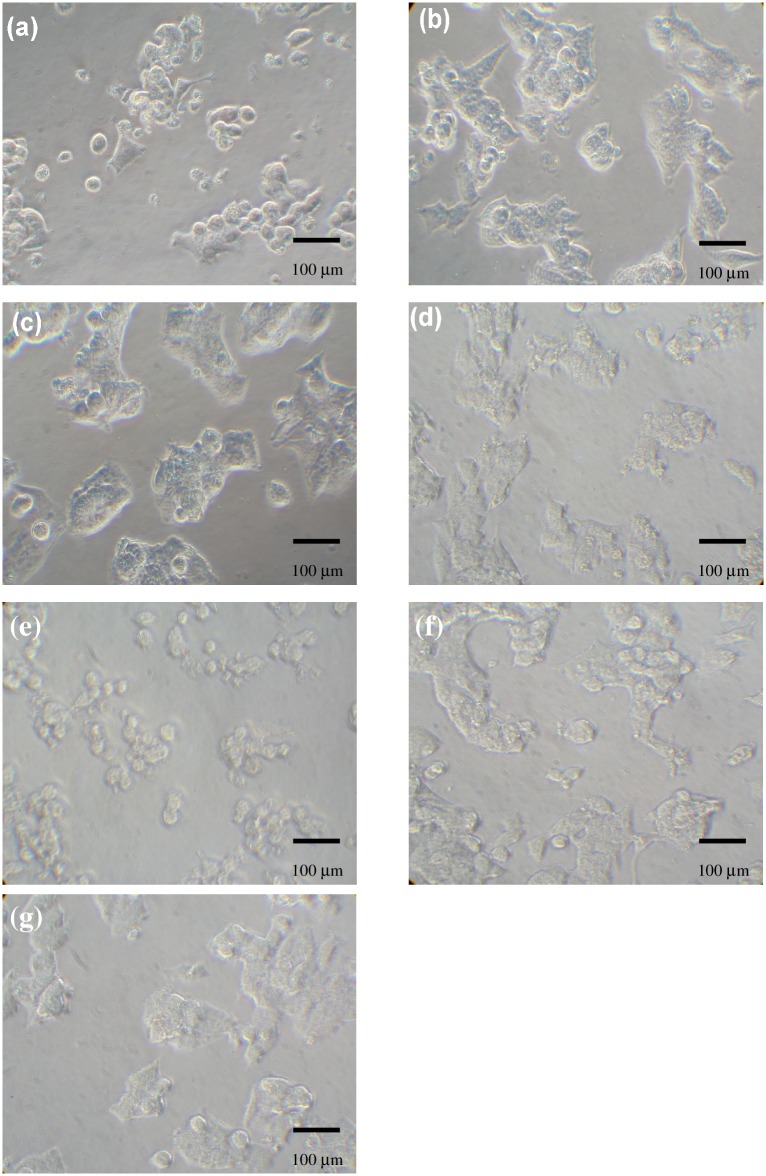
Morphology of Hep G2 cells treated with ACE/CS. Cells at a density of 2.0 × 10^5^ cells/well were incubated in medium containing (a) 21.2 μg/mL, (b) 10.6 μg/mL, (c) 5.3 μg/mL, and (d) 2.65 μg/mL ACE/CS for 48 h and observed by phase contrast microscopy (200×). Cells incubated with 100 μg/mL Trail (e) and without treatment (f) were defined as positive controls and controls, respectively. The nanoparticles without ACE polysaccharides (g), CSNP (667 μg/mL) were also examined. Each experiment (n = 3) was repeated 3 times independently to ensure reproducibility. ACE/CS: ACE polysaccharides encapsulated by chitosan-silica nanoparticles; ACE: *A*. *camphorata* extract; CSNP: chitosan-silica nanoparticles.

**Fig 3:**
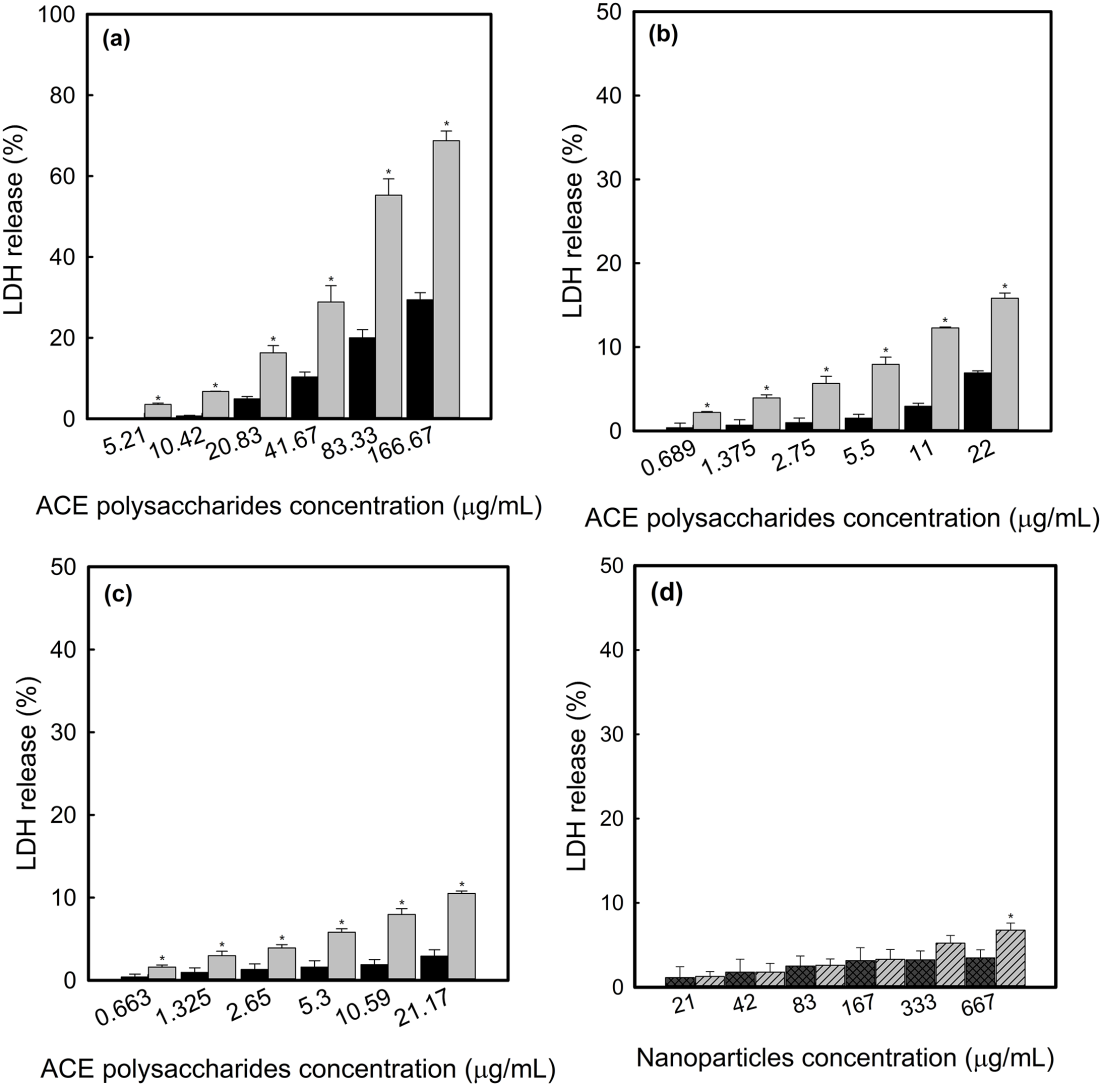
The LDH release of Hep G2 cells as incubated with (a) ACE polysaccharides, (b) ACE/CS, (c) ACE/S for (■) 24 h and (■) 48 h. Nanoparticles without ACE polysaccharides, CSNP (**▩**) and SNP (▨) were also examined at 48 h (d). Hep G2 cells were seeded in 96-well plates at a density of 2.0 × 10^5^ cells/well. After different treatments and processing, the resulting absorbance was measured at 490 nm and experiments were repeated 3 times independently to ensure reproducibility. Data with standard deviation of the mean are represented as error bars (n = 3) and values with asterisks were significantly different (*p* < 0.05) between 24 h and 48 h in (a-c), CSNP and SNP (d) at the corresponding concentrations. ACE: *A*. *camphorata* extract; ACE/CS: ACE polysaccharides encapsulated by chitosan-silica nanoparticles; ACE/S: ACE polysaccharides encapsulated by silica nanoparticles; CSNP: chitosan-silica nanoparticles; SNP: silica nanoparticles.

The death of Hep G2 cells was induced by ACE polysaccharides, ACE/CS and ACE/S in a dose and time-dependent manner (Fig 1). The results showed a slight decrease in cell viability at 24 h but became very significant after 48 h incubation, especially in the ACE/CS group (Fig 1b). Meanwhile, the viability of Hep G2 cells only decreased to approximately 85% in chitosan-silica nanoparticle (CSNP) or silica nanoparticle (SNP) treatments, which without ACE polysaccharides were loaded even at the highest concentration of 667 μg/mL for 48 h incubation (Fig 1c). Regarding morphology, cell shrinkage was obviously observed in phase-contract micrographs when exposed to ACE/CS (Fig 2a–2d) and a positive control (Fig 2e), but was insignificant in the CSNP treatment (Fig 2g). The effects of ACE polysaccharides and synthesized nanoparticles on Hep G2 cell membrane integrity, determined by a LDH assay, are shown in Fig 3a–3c. ACE polysaccharides, ACE/CS and ACE/S caused cell membrane damage in a dose- and time-dependent manner, especially at the highest concentration of 166.67 μg/mL ACE polysaccharide treatments for 48 h (> 60% LDH released). At the corresponding concentration; e.g., 166.67 μg/mL, ACE/ES and ACE/S (containing 13.2 μg/mL and 21.2 μg/mL ACE polysaccharides, respectively) showed much less membrane damage (< 20% LDH released; Fig 3b and 3c). The effects of nanoparticles without ACE polysaccharides on Hep G2 cell membrane integrity are shown in Fig 3d. None of the chitosan-silica composite treatments made a significant membranolytic effect up to 48 h even at a concentration of 667 μg/mL (< 3.7% LDH released). In contrast, silica nanoparticles were found to cause slight (< 6.5% LDH released) membrane damage in the same condition.

### ACE polysaccharides, ACE/CS and ACE/S caused DNA fragmentation and apoptosis

**Fig 4:**
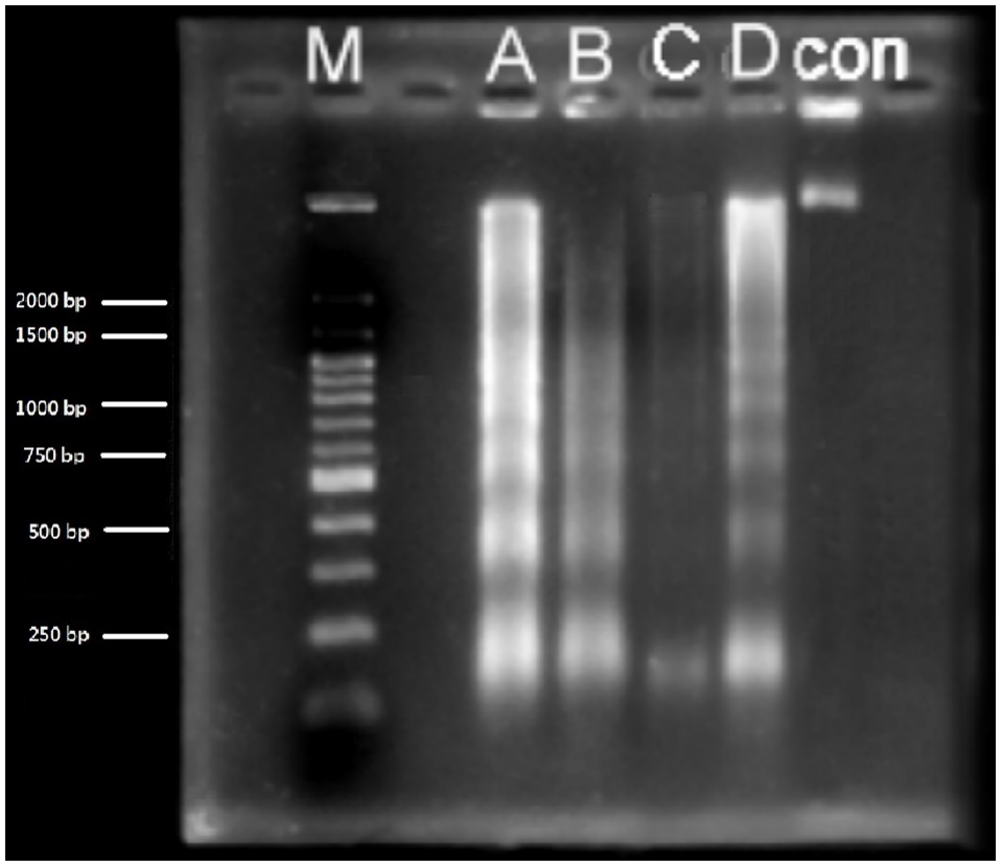
The DNA electrophoresis of Hep G2 cells after treatment with ACE polysaccharides, ACE/CS and ACE/S for 48 h. Hep G2 cells were seeded at the density of 2.0 × 10^5^ cells/mL in 10-cm dishes. After different treatments, cells were harvested, lysed and centrifuged at 14,000× *g* for supernatant collection. The fragmented DNAs were extracted, analyzed with 2% agarose gel electrophoresis containing 0.1 μg/mL ethidium bromide and imaged. Experiments were repeated 3 times independently to ensure reproducibility. M: Maker, A: 25 μg/mL ACE polysaccharides, B: ACE/CS (ACE polysaccharides = 13.2 μg/mL), C: ACE/S (ACE polysaccharides = 21.2 μg/mL), D: 100 μg/mL Trail for positive control and Con: without any treatment for control. ACE: *A*. *camphorata* extract; ACE/CS: ACE polysaccharides encapsulated by chitosan-silica nanoparticles; ACE/S: ACE polysaccharides encapsulated by silica nanoparticles.

The characteristic DNA laddering of apoptosis was carried out in Hep G2 cells treated with ACE polysaccharides, ACE/CS and ACE/S at the concentrations of 25 μg/mL, 13.2 μg/mL and 21.2 μg/mL, respectively, by using agarose gel electrophoresis. The results showed a ladder-like pattern of multiple DNA fragments consisting of approximately 180–200 base pairs (Fig 4). In cell cycle analysis, flow cytometry for quantifying the DNA fragmentation extent was used and the apoptotic cells were identified as the hypodiploidy peak (sub-G_1_) on the PI histogram. The percentage of apoptotic cells was 1.42% in the control group and reached 1.46% after treatment with ACE polysaccharides (25 μg/mL) in 24 h (Table 1 and S1 Fig). When cells were incubated with ACE/S (containing 21.2 μg/mL ACE polysaccharides) for 24 h, the sub-G_1_ phase population slightly increased to 2.76%, whereas it increased to 2.88% for the cells incubated in ACE/CS (containing 13.2 μg/mL ACE polysaccharides; Table 1 and S1 Fig). After 48 h incubation, the sub-G_1_ phase population approached 5.18% in the control group and reached 20.98% or 20.56% in ACE polysaccharides or ACE/CS group, respectively, but only increased to 10.86% for cells incubated with ACE/S (Table 1 and S1 Fig). In contrast, after Hep G2 was incubated with silica and chitosan-silica nanoparticles without ACE polysaccharides even at the highest concentration (667 μg/mL) for 48 h, the sub-G_1_ phase population was just slightly increased to 6.54% and 9.16% (Table 1).

**Table 1:** Cell cycle analysis for Hep G2 apoptosis induced by ACE polysaccharides, ACE/CS and ACE/S for 24 h and 48 h.

	Control	ACE polysaccharides	ACE/CS	ACE/S	CSNP	SNP
24 h	1.42±0.55^c^	1.46±0.21^c^	2.88±0.63^b^	2.76±0.45^b^	2.74±0.35^b^	3.82±0.66^a^
48 h	5.18±0.68^c^	20.98±1.13^a^	20.56±1.50^a^	10.86±1.15^b^	6.54±0.92^c^	9.16±0.60^b^

Hep G2 cells were seeded at the density of 1.0 × 10^6^ cells per 60-mm dish and treated with ACE polysaccharides (25 μg/mL), ACE/CS (ACE polysaccharides = 13.2 μg/mL) and ACE/S (ACE polysaccharides = 21.2 μg/mL). The nanoparticles without ACE polysaccharides (CSNP and SNP) were also examined. The cells without any treatment were defined as controls. Flow cytometry with excitation wavelength 488 nm/emission wavelength 630 nm was used for the analysis of PI-stained DNA, and data acquired from Cell Quest software based on a minimum of 10^5^ cells per sample were analyzed using a one-way ANOVA followed by Duncan’s test. The results are shown as the mean ± standard deviation (S.D.; n = 3), which without a common superscript (^a^, ^b^ and ^c^) at the same row were significantly different (*p*<0.05). ACE: *A*. *camphorata* extracts, ACE/CS: ACE polysaccharides encapsulated by chitosan-silica nanoparticles, ACE/S: ACE polysaccharides encapsulated by silica nanoparticles, CSNP: chitosan-silica nanoparticles, SNP: silica nanoparticles.

### ACE polysaccharides, ACE/CS and ACE/S decreased the ΔΨm

To investigate the Hep G2 cell mitochondrial apoptotic events induced by ACE polysaccharides, ACE/CS and ACE/S, we analyzed the values of the mitochondrial membrane potential (ΔΨm). As shown in Table 2 and S2 Fig, the ΔΨm decreased to 34.72%, 32.74% and 27.08% for the treatments of 48 h of ACE polysaccharides, ACE/CS and ACE/S, respectively. By comparison, the ΔΨm was only slightly decreased to 5.82% or 6.54% at the highest concentration (667 μg/mL) of SNP or CSNP treatment, respectively (Table 2 and S2 Fig).

**Table 2:** Effects of ACE polysaccharides and resulting nanoparticles on mitochondrial transmembrane potential, ROS, Fas and caspase activities in Hep G2 cells after 48 h incubation.

	ACE polysaccharides	ACE/CS	ACE/S	CSNP	SNP
Δφm	-34.72±0.63^d^	-32.74±0.38^c^	-27.08±0.90^b^	-5.82±1.13^a^	-6.54±1.16^a^
ROS	18.76±1.07^a^	13.36±0.74^b^	7.06±0.42^c^	1.86±0.69^d^	3.52±1.69^d^
Fas	47.64±0.74^a^	47.64±0.74^a^	47.64±0.74^a^	1.12±0.93^c^	15.02±1.82^b^
Caspase-8	38.74±2.23^a^	37.72±1.41^a^	28.58±1.68^b^	1.28±4.08^d^	18.98±1.07^c^
Caspase-9	41.42±1.64^a^	37.86±1.76^b^	27.24±1.84^c^	1.52±0.64^e^	5.86±0.53^d^
Caspase-3	40.34±1.36^a^	33.60±1.82^b^	19.24±1.40^c^	6.54±0.70^e^	13.02±0.38^d^

The nanoparticles without ACE polysaccharides (CSNP and SNP) were also examined. The values (%) are shown as sample (%)-control (%), where control was cells without any treatment. Data were analyzed using a one-way ANOVA followed by Duncan’s Test. The results are shown as the mean ± standard deviation (S.D.; n = 3), which without a common superscript (^a^, ^b^, ^c^, ^d^ and ^e^) at the same row were significantly different (*p*<0.05). Concentrations of samples used: ACE polysaccharides (25 μg/mL), ACE/CS (ACE polysaccharides = 13.2 μg/mL), ACE/S (ACE polysaccharides = 21.2 μg/mL), CSNP (667 μg/mL), SNP (667 μg/mL). ACE: *A*. *camphorata* extracts, ACE/CS: ACE polysaccharides encapsulated by chitosan-silica nanoparticles, ACE/S: ACE polysaccharides encapsulated by silica nanoparticles, CSNP: chitosan-silica nanoparticles, SNP: silica nanoparticles.

### ACE polysaccharides, ACE/CS and ACE/S increased the ROS generation

The DCF fluorescent response, which indicates the level of ROS, was increased significantly in Hep G2 cells after incubation with ACE polysaccharides, ACE/CS or ACE/S, as shown in Table 2 and S3 Fig. After 48 h of incubation, the ROS formation increased to 18.76% in the ACE polysaccharide treatment relative to controls and increased to 7.06% and 13.36% in ACE/CS and ACE/S treatments, respectively. The effects of SNP or CSNP on ROS generation are also shown in Table 2 and none of them caused significant ROS production (< 3.52%) in Hep G2 cells even at a concentration of 667 μg/mL for 48 h incubation.

### ACE polysaccharides, ACE/CS and ACE/S increased the expressions of Fas/APO-1 and caspase activities

The Fas/FasL system is the most important apoptosis initiator in cells and tissues. In this study, the expression of Fas/APO-1 was detected in Hep G2 cells after treatment with ACE polysaccharides, ACE/CS and ACE/S. The Fas/APO-1 content increased to 47.6% relative to controls after incubation with ACE polysaccharides, ACE/CS or ACE/S (Table 2 and S4 Fig). Meanwhile, the Fas/APO-1 content in Hep G2 cells was much lower when incubated with CSNP or SNP, which loaded without ACE polysaccharide (Table 2 and S4 Fig). Caspases are crucial mediators of programmed cell apoptosis. As shown in Table 2 and S5 Fig, the proteolytic activities of caspase-3, caspase-8 and caspase-9 in Hep G2 cells were increased up to 40% by treatment with ACE polysaccharides, ACE/CS and ACE/S. Unsurprisingly, CSNP and SNP caused less caspase activity than ACE polysaccharides and the resulting nanoparticles (Table 2 and S5 Fig).

## Discussion

In our previous study, the major composition of ACE was polysaccharides with a (1→3)-β-D-glucan structure, while the average particle sizes and encapsulation efficiencies of ACE/CS and ACE/S were 210 ± 13.3 nm and 294 ± 25.7 nm and 85.7% and 76.4%, respectively [28]. Many functions of polysaccharides extracted from *A*. *camphorate* have been identified [3, 10, 11]. In this study, we revealed ACE polysaccharides, ACE/CS and ACE/S all showed interesting cytotoxicity activities against Hep G2 cells, i.e., antitumor activity, especially with the ACE/CS treatment. Therefore, we further examined the cell morphology by phase-contrast microscopy in the ACE/CS group and cell shrinkage was obviously present. Apparently, the nano-encapsulation of ACE polysaccharides provided a viable approach for enhancing antitumor efficacy in liver cancer cells. Moreover, previous studies showed that polysaccharides extracted from *A*. *camphorata* had no cytotoxic effect on human leukemic U937 cells, human endothelial cells and normal hepatocytes cells [11]. Our earlier study also reported ACE polysaccharides, ACE/CS or ACE/S were nontoxic to three human normal cell lines; i.e., human skin fibroblast CCD-966sk cells, human normal fibroblast WS1 cells and human lung embryonic MRC-5 cells [28]. These studies suggested that AEC polysaccharides, ACE/CS and ACE/S are only toxic to cancer cells, such as Hep G2 cells, but not to normal cells. In contrast, the cytotoxicity of chitosan-silica or silica nanoparticles without ACE polysaccharides loaded on Hep G2 cells was insignificant, and this result was consistent with the results of our previous study, which demonstrated that silica nanoparticles became more toxic in normal human fibroblast cells than in cancer cells at high concentrations [25].

LDH leakage from cells is the explicit evidence of cell membrane damage and refers to one of the physiological features of necrotic cell death [29]. In the present study, we found LDH leakage was increased as the concentrations of ACE polysaccharides, ACE/CS and ACE/S treatments increased in the Hep G2 cells. These results were in agreement with the data obtained from the MTT assay (Fig 1). Meanwhile, 166.67 μg/mL of ACE/CS and ACE/S (containing 13.2 μg/mL and 21.2 μg/mL ACE polysaccharides, respectively) induced smaller membrane damage (< 20% LDH released) than the cytotoxicity (< 40% cell viability) released at the corresponding concentrations. The slight difference between cell viability and membrane damage could result from apoptosis and necrosis that existed concurrently when induced by nanoparticles [30, 31]. However, at the highest concentration (166.67 μg/mL), ACE polysaccharides caused a massive (> 60% LDH released) membranolytic effect on Hep G2 cells in 48 h; hence, we deduced that high concentrations of ACE polysaccharides may induce cell necrosis. Therefore, the concentrations of ACE polysaccharides in the following tests were maintained at the level below the cytotoxic effect to evaluate the apoptosis mechanisms in Hep G2 cells.

Fragmentation of DNA is one of the most important and irreversible events in apoptosis. To further examine cell apoptosis that was not affected by necrosis, DNA fragmentation and a cell cycle assay were performed in this study. The results revealed that ACE polysaccharides, ACE/CS or ACE/S treatments could induce DNA degradation and resulted in the appearance of a ladder-like pattern. The cell cycle study also showed that the ACE polysaccharides, ACE/S and ACE/S increased the sub-G_1_ phase population of Hep G2 cells in a time-dependent manner. Research has shown that *A*. *camphorata* can cause the accumulation of the sub-G_1_ population with hypodiploidic DNA content, which is a typical marker of apoptosis and triggers cell death [8]. The assays described above demonstrated that the ACE polysaccharides and resulting nanoparticles were capable of inducing apoptosis and triggering cell death.

In previous studies, *A*. *camphorata* could induce apoptosis in cancer cells by triggering the mitochondrial pathway [5]. In this study, the ΔΨm of Hep G2 cells decreased dramatically after 48 h of incubation with ACE polysaccharides, ACE/CS or ACE/S, which indicated that ACE polysaccharides and the resulting nanoparticles could induce the apoptosis of Hep G2 cells through damage of the mitochondrial membrane. Meanwhile, the factors of oxidative stress, such as ROS and lipid peroxidation, have been observed in some apoptotic processes [32–34]. ROS plays an important role in apoptosis by regulating the activity of certain enzymes involved in the cell death pathway. In our study, the incubation of Hep G2 cells with ACE polysaccharides, ACE/CS or ACE/S induced ROS generation and caused the subsequent apoptosis. However, a previous study revealed that *A*. *camphorata* might possess antioxidant properties against ROS generation [35]. This conflict showed the active components in *A*. *camphorata* might serve as mediators of the reactive oxygen scavenging system and have the potential to act as both pro-oxidants and antioxidants, depending on the redox state of the biological environment.

The Fas/FasL system is a key signaling transduction pathway of apoptosis in cells and tissues [36, 37]. Many publications have highlighted the role of the Fas/FasL system in chemotherapy-induced apoptosis of tumors by up-regulating Fas/APO-1 or FasL [38]. In this study, we found Fas/APO-1 expression in Hep G2 cells dramatically increased after incubation with ACE polysaccharides, ACE/CS or ACE/S, meaning those materials have the ability to induce Fas-mediated apoptosis. In the case of caspases, the proteolytic activities of caspase-3, caspase-8, and caspase-9 on Hep G2 cells increased from 19% to nearly 42% after ACE polysaccharides, ACE/CS or ACE/S incubation for 48 h. Activated caspase-8 and caspase-9 further initiated the activation of the caspase cascade leading to biochemical and morphological changes associated with apoptosis [39–41]. Caspase-3 is a well-known downstream effector of caspase that can be activated by caspase-8 or caspase-9 via different signaling pathways [42]. The results of our study demonstrated that ACE polysaccharides, ACE/CS and ACE/S activated the caspase cascade and resulted in Hep G2 cell death.

The results of the apoptosis assays shown above revealed that ACE polysaccharides, ACE/CS or ACE/S induced similar apoptosis mechanisms in the Hep G2 cells, but it took a smaller dose of ACE/CS (ACE polysaccharides = 13.2 μg/mL) to reach the same effect than in ACE/S (ACE polysaccharides = 21.2 μg/mL) and ACE polysaccharides (25 μg/mL). This result suggested that nano-encapsulation increased the apoptosis effect of ACE polysaccharides in the Hep G2 cells, and ACE/CS could have more potential than ACE/S. In conclusion, ACE polysaccharides, ACE/CS and ACE/S appear to be promising antitumor agents for liver cancer cells. The encapsulation of ACE polysaccharides by chitosan-silica nanoparticles might provide a useful carrier for antitumor ingredients.

## Supporting Information

S1 Fig Cell cycle analysis for Hep G2 apoptosis induced by ACE polysaccharides, ACE/CS and ACE/S for 24 h and 48 h. Hep G2 cells were seeded at the density of 1.0 × 10^6^ cells per 60-mm dish and treated with (b) ACE polysaccharides (25 μg/mL), (c) ACE/CS (ACE polysaccharides = 13.2 μg/mL) and (d) ACE/S (ACE polysaccharides = 21.2 μg/mL). Cells without any treatment were defined as controls (a). A flow cytometer with an excitation wavelength 488 nm and an emission wavelength 630 nm was used for the analysis of PI-stained DNA, and data were acquired using Cell Quest software based on a minimum of 10^5^ cells per sample. Experiments were repeated 3 times independently to ensure reproducibility, and data were acquired in triplicate (n = 3). ACE: *A*. *camphorata* extract; ACE/CS: ACE polysaccharides encapsulated by chitosan-silica nanoparticles; ACE/S: ACE polysaccharides encapsulated by silica nanoparticles
(PDF)

S2 Fig The effects of ACE polysaccharides, ACE/CS and ACE/S incubated for 48 h on the mitochondrial transmembrane potential in Hep G2 cells. After different treatments and re-suspension at a density of 1.0 × 10^6^ cells/mL in PBS, the cells were stained with 25 μM Rhodamine 123 and the membrane potential (ΔΨ) was detected with a flow cytometer with excitation at 488 nm and emission at 520 nm based on a minimum of 10^5^ cells per sample. The different treatments were represented as (a) controls, (b) ACE polysaccharides (25 μg/mL), (c) ACE/CS (ACE polysaccharides = 13.2 μg/mL) and (d) ACE/S (ACE polysaccharides = 21.2 μg/mL). The nanoparticles without ACE polysaccharides (e) SNP (667 μg/mL) and (f) CSNP (667 μg/mL) were also examined. Experiments were repeated 3 times independently to ensure reproducibility and data were acquired in triplicate (n = 3). ACE: *A*. *camphorata* extract; ACE/CS: ACE polysaccharides encapsulated by chitosan-silica nanoparticles; ACE/S: ACE polysaccharides encapsulated by silica nanoparticles; CSNP: chitosan-silica nanoparticles; SNP: silica nanoparticles
(PDF)

S3 Fig The ROS generation in Hep G2 cells induced for 48 h by ACE polysaccharides, ACE/CS and ACE/S. After different treatments and trypsinization, the cells were washed, re-suspended at a density of 1.0 × 10^6^ cells/mL in PBS and then kept in a dark chamber containing DCFDA (20 μM) for further flow cytometric analysis (488 nm excitation/520 nm emission). The different treatments were expressed as (a) controls, (b) ACE polysaccharides (25 μg/mL), (c) ACE/CS (ACE polysaccharides = 13.2 μg/mL) and (d) ACE/S (ACE polysaccharides = 21.2 μg/mL). The nanoparticles without ACE polysaccharides (e) SNP (667 μg/mL) and (f) CSNP (667 μg/mL) were also examined. Experiments were repeated 3 times independently to ensure reproducibility and data were acquired in triplicate (n = 3). ACE: *A*. *camphorata* extract; ACE/CS: ACE polysaccharides encapsulated by chitosan-silica nanoparticles; ACE/S: ACE polysaccharides encapsulated by silica nanoparticles; CSNP: chitosan-silica nanoparticles; SNP: silica nanoparticles
(PDF)

S4 Fig The Fas/APO-1 expression of Hep G2 cells stimulated by ACE polysaccharides, ACE/CS and ACE/S for 48 h. Hep G2 cells with different treatments, trypsinization, washing and re-suspending at a density of 1.0 × 10^6^ cells/mL in PBS and incubated with phycoerythin (PE)-conjugated Fas monoclonal antibody UB2 (1 pg/mL). Samples were then valuated by flow cytometry with excitation at 488 nm and emission at 580 nm and data were acquired using Cell Quest software based on a minimum of 10^5^ cells per sample. The different treatments were represented as (a) controls, (b) ACE polysaccharides (25 μg/mL), (c) ACE/CS (ACE polysaccharides = 13.2 μg/mL) and (d) ACE/S (ACE polysaccharides = 21.2 μg/mL). The nanoparticles without ACE polysaccharides (e) SNP (667 μg/mL) and (f) CSNP (667 μg/mL) were also examined. Experiments were repeated 3 times independently to ensure reproducibility and data were acquired in triplicate (n = 3). ACE: *A*. *camphorata* extract; ACE/CS: ACE polysaccharides encapsulated by chitosan-silica nanoparticles; ACE/S: ACE polysaccharides encapsulated by silica nanoparticles; CSNP: chitosan-silica nanoparticles; SNP: silica nanoparticles
(PDF)

S5 Fig The effects of ACE polysaccharides, ACE/CS and ACE/S on caspase 9, caspase 8 and caspase 3 in Hep G2 cells for 48 h incubation. Cells were pre-incubated with different treatments and counted to 1.0 × 10^6^ cells/60-mm dish. After centrifuging, the cell pellets were lysed and supernatants were collected. DEVD-pNA, IETD-pNA or LEHD-pNA was added for a substrate and fluorescence was detected by flow cytometry with excitation at 485 nm and emission at 520 nm based on a minimum of 10^5^ cells per sample. The different treatments were represented as (a) controls, (b) ACE polysaccharides (25 μg/mL), (c) ACE/CS (ACE polysaccharides = 13.2 μg/mL) and (d) ACE/S (ACE polysaccharides = 21.2 μg/mL). The nanoparticles without ACE polysaccharides (e) SNP (667 μg/mL) and (f) CSNP (667 μg/mL) were also examined. Experiments were repeated 3 times independently to ensure reproducibility and data were acquired in triplicate (n = 3). ACE: *A*. *camphorata* extract; ACE/CS: ACE polysaccharides encapsulated by chitosan-silica nanoparticles; ACE/S: ACE polysaccharides encapsulated by silica nanoparticles; CSNP: chitosan-silica nanoparticles; SNP: silica nanoparticles.
(PDF)

## References

[1] Borchers AT, Keen CL, Gershwin ME. Mushrooms, tumors, and immunity: an update. Experimental Biology and Medicine. 2004;229(5):393–406. 15096651 10.1177/153537020422900507

[2] Borchers AT, Krishnamurthy A, Keen CL, Meyers FJ, Gershwin ME. The immunobiology of mushrooms. Experimental biology and medicine. 2008;233(3):259–76. 10.3181/0708-MR-227 18296732

[3] Geethangili M, Tzeng Y-M. Review of pharmacological effects of Antrodia camphorata and its bioactive compounds. Evidence-Based Complementary and Alternative Medicine. 2011;2011. 10.1093/ecam/nep108 PMC3095428 19687189

[4] Wang G-J, Tseng H-W, Chou C-J, Tsai T-H, Chen C-T, Lu M-K. The vasorelaxation of Antrodia camphorata mycelia: involvement of endothelial Ca 2+-NO-cGMP pathway. Life sciences. 2003;73(21):2769–83. 13679244 10.1016/s0024-3205(03)00669-6

[5] Hsiao G, Shen M-Y, Lin K-H, Lan M-H, Wu L-Y, Chou D-S, et al Antioxidative and hepatoprotective effects of Antrodia camphorata extract. Journal of Agricultural and Food Chemistry. 2003;51(11):3302–8. 12744658 10.1021/jf021159t

[6] Song T-Y, Yen G-C. Protective effects of fermented filtrate from Antrodia camphorata in submerged culture against CCl4-induced hepatic toxicity in rats. Journal of Agricultural and Food Chemistry. 2003;51(6):1571–7. 12617586 10.1021/jf0209701

[7] Hsu Y-L, Kuo Y-C, Kuo P-L, Ng L-T, Kuo Y-H, Lin C-C. Apoptotic effects of extract from Antrodia camphorata fruiting bodies in human hepatocellular carcinoma cell lines. Cancer letters. 2005;221(1):77–89. 15797630 10.1016/j.canlet.2004.08.012

[8] Yang H-L, Chen C-S, Chang W-H, Lu F-J, Lai Y-C, Chen C-C, et al Growth inhibition and induction of apoptosis in MCF-7 breast cancer cells by Antrodia camphorata. Cancer letters. 2006;231(2):215–27. 16399223 10.1016/j.canlet.2005.02.004

[9] Peng C-C, Chen K-C, Peng RY, Chyau C-C, Su C-H, Hsieh-Li HM. Antrodia camphorata extract induces replicative senescence in superficial TCC, and inhibits the absolute migration capability in invasive bladder carcinoma cells. Journal of ethnopharmacology. 2007;109(1):93–103. 16930895 10.1016/j.jep.2006.07.009

[10] Lee I-H, Huang R-L, Chen C-T, Chen H-C, Hsu W-C, Lu M-K. Antrodia camphorata polysaccharides exhibit anti-hepatitis B virus effects. FEMS Microbiology Letters. 2002;209(1):63–7. 12007655 10.1111/j.1574-6968.2002.tb11110.x

[11] Liu J-J, Huang T-S, Hsu M-L, Chen C-C, Lin W-S, Lu F-J, et al Antitumor effects of the partially purified polysaccharides from Antrodia camphorata and the mechanism of its action. Toxicology and applied pharmacology. 2004;201(2):186–93. 15541758 10.1016/j.taap.2004.05.016

[12] Wang X, Yang L, Chen ZG, Shin DM. Application of nanotechnology in cancer therapy and imaging. CA: a cancer journal for clinicians. 2008;58(2):97–110. 18227410 10.3322/CA.2007.0003

[13] Kumar MR, Muzzarelli RA, Muzzarelli C, Sashiwa H, Domb A. Chitosan chemistry and pharmaceutical perspectives. Chemical reviews. 2004;104(12):6017–84. 15584695 10.1021/cr030441b

[14] Bodnar M, Hartmann JF, Borbely J. Preparation and characterization of chitosan-based nanoparticles. Biomacromolecules. 2005;6(5):2521–7. 16153088 10.1021/bm0502258

[15] Janes KA, Fresneau MP, Marazuela A, Fabra A, Alonso MJ. Chitosan nanoparticles as delivery systems for doxorubicin. Journal of Controlled Release. 2001;73(2):255–67. 11516503 10.1016/s0168-3659(01)00294-2

[16] Lozano MV, Torrecilla D, Torres D, Vidal A, Domiínguez F, Alonso MJ. Highly efficient system to deliver taxanes into tumor cells: docetaxel-loaded chitosan oligomer colloidal carriers. Biomacromolecules. 2008;9(8):2186–93. 10.1021/bm800298u 18637687

[17] Mi F-L, Wu Y-Y, Chiu Y-L, Chen M-C, Sung H-W, Yu S-H, et al Synthesis of a novel glycoconjugated chitosan and preparation of its derived nanoparticles for targeting HepG2 cells. Biomacromolecules. 2007;8(3):892–8. 17316043 10.1021/bm060998b

[18] Zhang H, Oh M, Allen C, Kumacheva E. Monodisperse chitosan nanoparticles for mucosal drug delivery. Biomacromolecules. 2004;5(6):2461–8. 15530064 10.1021/bm0496211

[19] Cai X-J, Xu Y-Y. Nanomaterials in controlled drug release. Cytotechnology. 2011;63(4):319–23. 10.1007/s10616-011-9366-5 21720796 PMC3140842

[20] Lin L-N, Liu Q, Song L, Liu F-F, Sha J-X. Recent advances in nanotechnology based drug delivery to the brain. Cytotechnology. 2010;62(5):377–80. 10.1007/s10616-010-9295-8 20700653 PMC2993862

[21] Barbe C, Bartlett J, Kong L, Finnie K, Lin HQ, Larkin M, et al Silica Particles: A Novel Drug-Delivery System. Advanced materials. 2004;16(21):1959–66.

[22] Slowing II, Trewyn BG, Giri S, Lin VY. Mesoporous silica nanoparticles for drug delivery and biosensing applications. Advanced Functional Materials. 2007;17(8):1225–36.

[23] Coradin T, Lopez PJ. Biogenic silica patterning: simple chemistry or subtle biology? ChemBioChem. 2003;4(4):251–9. 12672103 10.1002/cbic.200390044

[24] Chang J-S, Kong Z-L, Hwang D-F, Chang KLB. Chitosan-catalyzed aggregation during the biomimetic synthesis of silica nanoparticles. Chemistry of materials. 2006;18(3):702–7.

[25] Chang J-S, Chang KLB, Hwang D-F, Kong Z-L. In vitro cytotoxicitiy of silica nanoparticles at high concentrations strongly depends on the metabolic activity type of the cell line. Environmental Science & Technology. 2007;41(6):2064–8. 17410806 10.1021/es062347t

[26] Mosmann T. Rapid colorimetric assay for cellular growth and survival: application to proliferation and cytotoxicity assays. Journal of immunological methods. 1983;65(1):55–63. 6606682 10.1016/0022-1759(83)90303-4

[27] Chou C-T, Lin W-F, Kong Z-L, Chen S-Y, Hwang D-F. Taurine prevented cell cycle arrest and restored neurotrophic gene expression in arsenite-treated SH-SY5Y cells. Amino acids. 2013;45(4):811–9. 10.1007/s00726-013-1524-y 23744399

[28] Kong Z-L, Chang J-S, Chang KLB. Antiproliferative effect of Antrodia camphorata polysaccharides encapsulated in chitosan–silica nanoparticles strongly depends on the metabolic activity type of the cell line. Journal of nanoparticle research. 2013;15(9):1–13.

[29] Hardman R. A toxicologic review of quantum dots: toxicity depends on physicochemical and environmental factors. Environmental health perspectives. 2006;114(2):165–72. 16451849 10.1289/ehp.8284 PMC1367826

[30] Shim W, Paik MJ, Nguyen D-T, Lee J-K, Lee Y, Kim J-H, et al Analysis of Changes in Gene Expression and Metabolic Profiles Induced by Silica-Coated Magnetic Nanoparticles. ACS nano. 2012;6(9):7665–80. 22830605 10.1021/nn301113f

[31] Foldbjerg R, Olesen P, Hougaard M, Dang DA, Hoffmann HJ, Autrup H. PVP-coated silver nanoparticles and silver ions induce reactive oxygen species, apoptosis and necrosis in THP-1 monocytes. Toxicology Letters. 2009;190(2):156–62. 10.1016/j.toxlet.2009.07.009 19607894

[32] Latchoumycandane C, Marathe GK, Zhang R, McIntyre TM. Oxidatively truncated phospholipids are required agents of tumor necrosis factor α (TNFα)-induced apoptosis. Journal of Biological Chemistry. 2012;287(21):17693–705. 10.1074/jbc.M111.300012 22433871 PMC3366783

[33] Maes M, Fišar Z, Medina M, Scapagnini G, Nowak G, Berk M. New drug targets in depression: inflammatory, cell-mediated immune, oxidative and nitrosative stress, mitochondrial, antioxidant, and neuroprogressive pathways. And new drug candidates—Nrf2 activators and GSK-3 inhibitors. Inflammopharmacology. 2012;20(3):127–50. 10.1007/s10787-011-0111-7 22271002

[34] Silva ACRA, de Almeida BFM, Soeiro CS, Ferreira WL, de Lima VMF, Ciarlini PC. Oxidative stress, superoxide production, and apoptosis of neutrophils in dogs with chronic kidney disease. Canadian Journal of Veterinary Research. 2013;77(2):136–41. 24082406 PMC3605930

[35] Huang G-J, Deng J-S, Huang S-S, Shao Y-Y, Chen C-C, Kuo Y-H. Protective effect of antrosterol from Antrodia camphorata submerged whole broth against carbon tetrachloride-induced acute liver injury in mice. Food Chemistry. 2012;132(2):709–16.

[36] Mou H, Zheng Y, Zhao P, Bao H, Fang W, Xu N. Celastrol induces apoptosis in non-small-cell lung cancer A549 cells through activation of mitochondria-and Fas/FasL-mediated pathways. Toxicology in Vitro. 2011;25(5):1027–32. 10.1016/j.tiv.2011.03.023 21466843

[37] Nagata S, Golstein P. The Fas death factor. Science. 1995;267(5203):1449–56. 7533326 10.1126/science.7533326

[38] Hseu Y-C, Yang H-L, Lai Y-C, Lin J-G, Chen G-W, Chang Y-H. Induction of apoptosis by Antrodia camphorata in human premyelocytic leukemia HL-60 cells. Nutrition and Cancer. 2004;48(2):189–97. 15231454 10.1207/s15327914nc4802_9

[39] Li P, Nijhawan D, Budihardjo I, Srinivasula SM, Ahmad M, Alnemri ES, et al Cytochrome c and dATP-dependent formation of Apaf-1/caspase-9 complex initiates an apoptotic protease cascade. Cell. 1997;91(4):479–89. 9390557 10.1016/s0092-8674(00)80434-1

[40] Mignotte B, Vayssiere JL. Mitochondria and apoptosis. European Journal of Biochemistry. 1998;252(1):1–15. 9523706 10.1046/j.1432-1327.1998.2520001.x

[41] Tepper CG, Seldin MF, Mudryj M. Fas-mediated apoptosis of proliferating, transiently growth-arrested, and senescent normal human fibroblasts. Experimental cell research. 2000;260(1):9–19. 11010806 10.1006/excr.2000.4990

[42] Cohen G. Caspases: the executioners of apoptosis. Biochem j. 1997;326:1–16. 9337844 10.1042/bj3260001 PMC1218630

