# Supplementary material for: Apoptosis of Hepatocellular Carcinoma Cells Induced by Nanoencapsulated Polysaccharides Extracted from Antrodia Camphorata
Source: PLoS One. 2015 Sep 1;10(9):e0136782. doi: 10.1371/journal.pone.0136782 (PMC4556685; doi:10.1371/journal.pone.0136782)
Supplement: S1 Fig — Hep G2 cells were seeded at the density of 1.0 × 10^6^ cells per 60-mm dish and treated with (b) ACE polysaccharides (25 μg/mL), (c) ACE/CS (ACE polysaccharides = 13.2 μg/mL) and (d) ACE/S (ACE polysaccharides = 21.2 μg/mL). Cells without any treatment were defined as controls (a). A flow cytometer with an excitation wavelength 488 nm and an emission wavelength 630 nm was used for the analysis of PI-stained DNA, and data were acquired using Cell Quest software based on a minimum of 105 cells per sample. Experiments were repeated 3 times independently to ensure reproducibility, and data were acquired in triplicate (n = 3). ACE: A. camphorata extract; ACE/CS: ACE polysaccharides encapsulated by chitosan-silica nanoparticles; ACE/S: ACE polysaccharides encapsulated by silica nanoparticles (PDF) [file pone.0136782.s001.pdf]

(A)

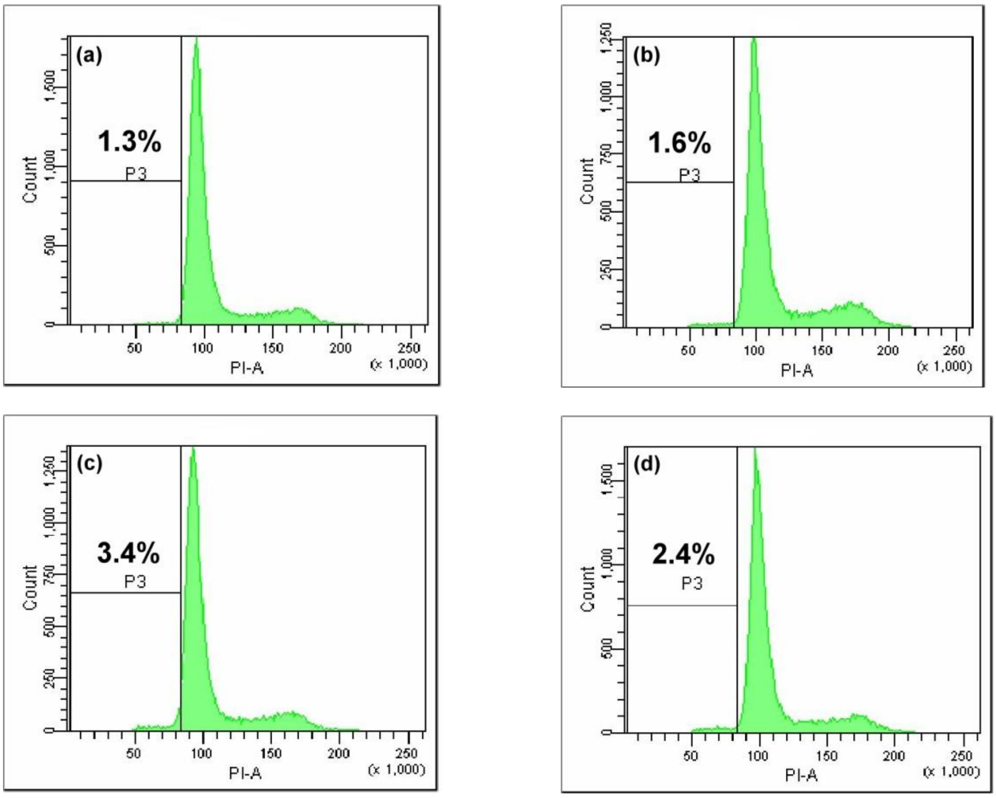
(a)
1.3%
P3
(b)
1.6%
P3
(c)
3.4%
P3
(d)
2.4%
P3
Count
PI-A
(x 1,000)

(B)

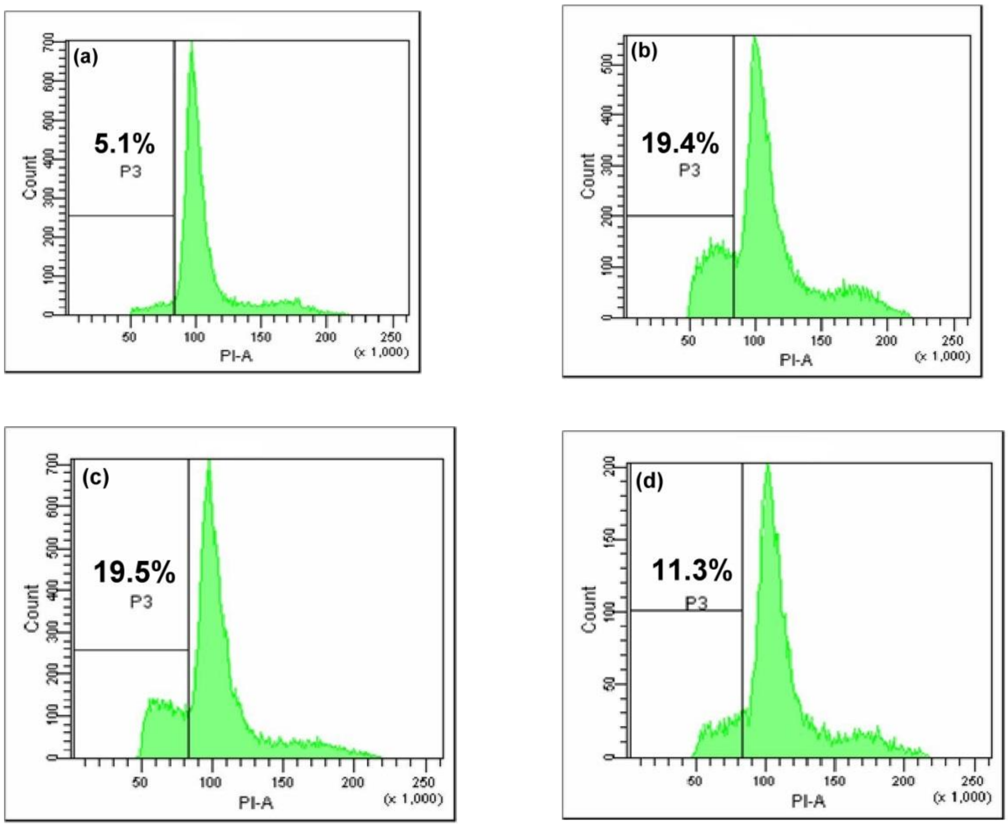
(a)
5.1%
P3
(b)
19.4%
P3
(c)
19.5%
P3
(d)
11.3%
P3
Count
PI-A
(x 1,000)

Fig. S1. Cell cycle analysis of Hep G2 apoptosis induced by ACE polysaccharides, ACE/CS and ACE/S for (A) 24 and (B) 48 h
