# Supplementary figures and images for: Apoptosis of Hepatocellular Carcinoma Cells Induced by Nanoencapsulated Polysaccharides Extracted from Antrodia Camphorata
Source: PLoS One. 2015 Sep 1;10(9):e0136782. doi: 10.1371/journal.pone.0136782 (PMC4556685; doi:10.1371/journal.pone.0136782)

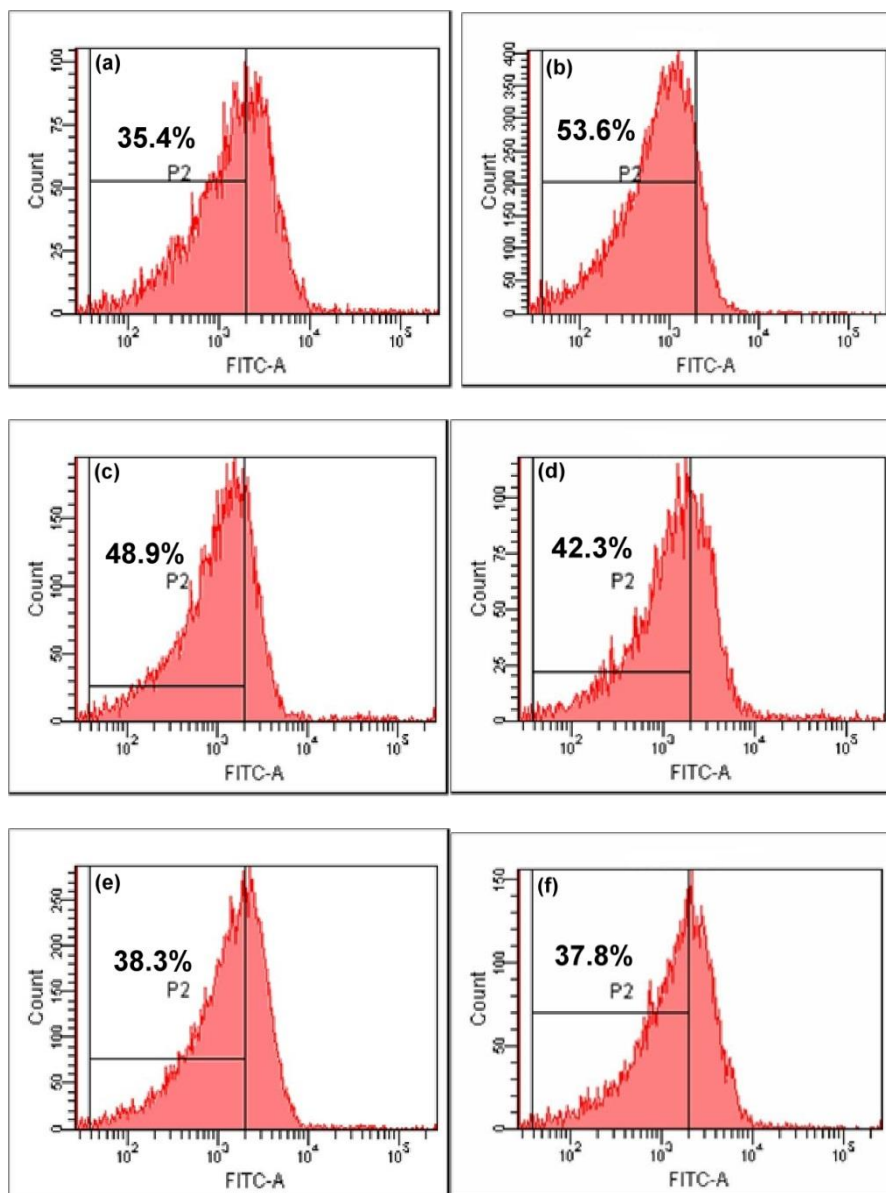

**Fig. S3. The ROS generation in Hep G2 cells induced for 48 h by ACE**

**polysaccharides, ACE/CS and ACE/S.**

Supplement: S3 Fig — After different treatments and trypsinization, the cells were washed, re-suspended at a density of 1.0 × 10^6^ cells/mL in PBS and then kept in a dark chamber containing DCFDA (20 μM) for further flow cytometric analysis (488 nm excitation/520 nm emission). The different treatments were expressed as (a) controls, (b) ACE polysaccharides (25 μg/mL), (c) ACE/CS (ACE polysaccharides = 13.2 μg/mL) and (d) ACE/S (ACE polysaccharides = 21.2 μg/mL). The nanoparticles without ACE polysaccharides (e) SNP (667 μg/mL) and (f) CSNP (667 μg/mL) were also examined. Experiments were repeated 3 times independently to ensure reproducibility and data were acquired in triplicate (n = 3). ACE: A. camphorata extract; ACE/CS: ACE polysaccharides encapsulated by chitosan-silica nanoparticles; ACE/S: ACE polysaccharides encapsulated by silica nanoparticles; CSNP: chitosan-silica nanoparticles; SNP: silica nanoparticles (PDF) [file pone.0136782.s003.pdf]
