# Supplementary material for: Apoptosis of Hepatocellular Carcinoma Cells Induced by Nanoencapsulated Polysaccharides Extracted from Antrodia Camphorata
Source: PLoS One. 2015 Sep 1;10(9):e0136782. doi: 10.1371/journal.pone.0136782 (PMC4556685; doi:10.1371/journal.pone.0136782)
Supplement: S4 Fig — Hep G2 cells with different treatments, trypsinization, washing and re-suspending at a density of 1.0 × 10^6^ cells/mL in PBS and incubated with phycoerythin (PE)-conjugated Fas monoclonal antibody UB2 (1 pg/mL). Samples were then valuated by flow cytometry with excitation at 488 nm and emission at 580 nm and data were acquired using Cell Quest software based on a minimum of 105 cells per sample. The different treatments were represented as (a) controls, (b) ACE polysaccharides (25 μg/mL), (c) ACE/CS (ACE polysaccharides = 13.2 μg/mL) and (d) ACE/S (ACE polysaccharides = 21.2 μg/mL). The nanoparticles without ACE polysaccharides (e) SNP (667 μg/mL) and (f) CSNP (667 μg/mL) were also examined. Experiments were repeated 3 times independently to ensure reproducibility and data were acquired in triplicate (n = 3). ACE: A. camphorata extract; ACE/CS: ACE polysaccharides encapsulated by chitosan-silica nanoparticles; ACE/S: ACE polysaccharides encapsulated by silica nanoparticles; CSNP: chitosan-silica nanoparticles; SNP: silica nanoparticles (PDF) [file pone.0136782.s004.pdf]

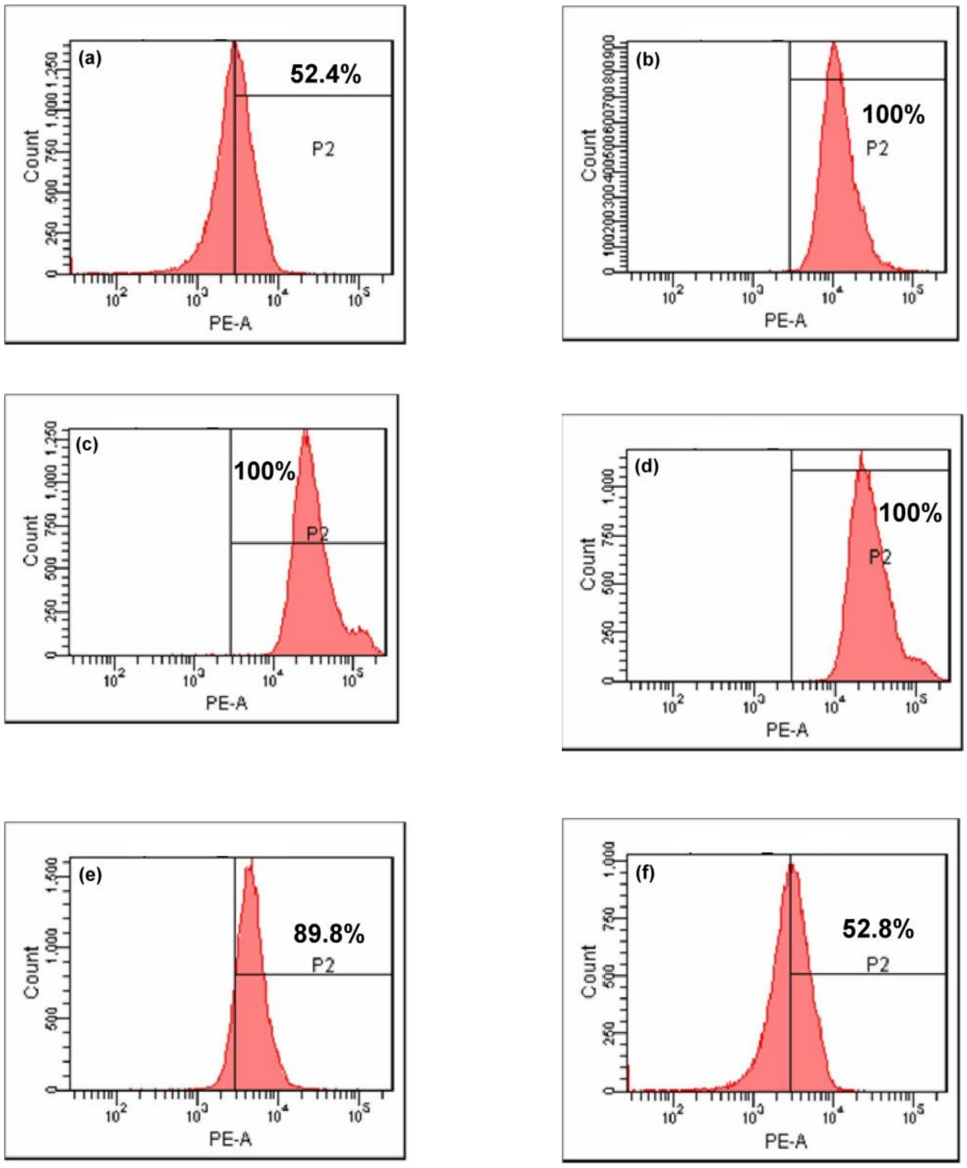

Fig. S4. The Fas/APO-1 expression of Hep G2 cells stimulated by ACE polysaccharides, ACE/CS and ACE/S for 48 h.
