# Supplementary material for: Apoptosis of Hepatocellular Carcinoma Cells Induced by Nanoencapsulated Polysaccharides Extracted from Antrodia Camphorata
Source: PLoS One. 2015 Sep 1;10(9):e0136782. doi: 10.1371/journal.pone.0136782 (PMC4556685; doi:10.1371/journal.pone.0136782)
Supplement: S5 Fig — Cells were pre-incubated with different treatments and counted to 1.0 × 10^6^ cells/60-mm dish. After centrifuging, the cell pellets were lysed and supernatants were collected. DEVD-pNA, IETD-pNA or LEHD-pNA was added for a substrate and fluorescence was detected by flow cytometry with excitation at 485 nm and emission at 520 nm based on a minimum of 105 cells per sample. The different treatments were represented as (a) controls, (b) ACE polysaccharides (25 μg/mL), (c) ACE/CS (ACE polysaccharides = 13.2 μg/mL) and (d) ACE/S (ACE polysaccharides = 21.2 μg/mL). The nanoparticles without ACE polysaccharides (e) SNP (667 μg/mL) and (f) CSNP (667 μg/mL) were also examined. Experiments were repeated 3 times independently to ensure reproducibility and data were acquired in triplicate (n = 3). ACE: A. camphorata extract; ACE/CS: ACE polysaccharides encapsulated by chitosan-silica nanoparticles; ACE/S: ACE polysaccharides encapsulated by silica nanoparticles; CSNP: chitosan-silica nanoparticles; SNP: silica nanoparticles. (PDF) [file pone.0136782.s005.pdf]

(A)

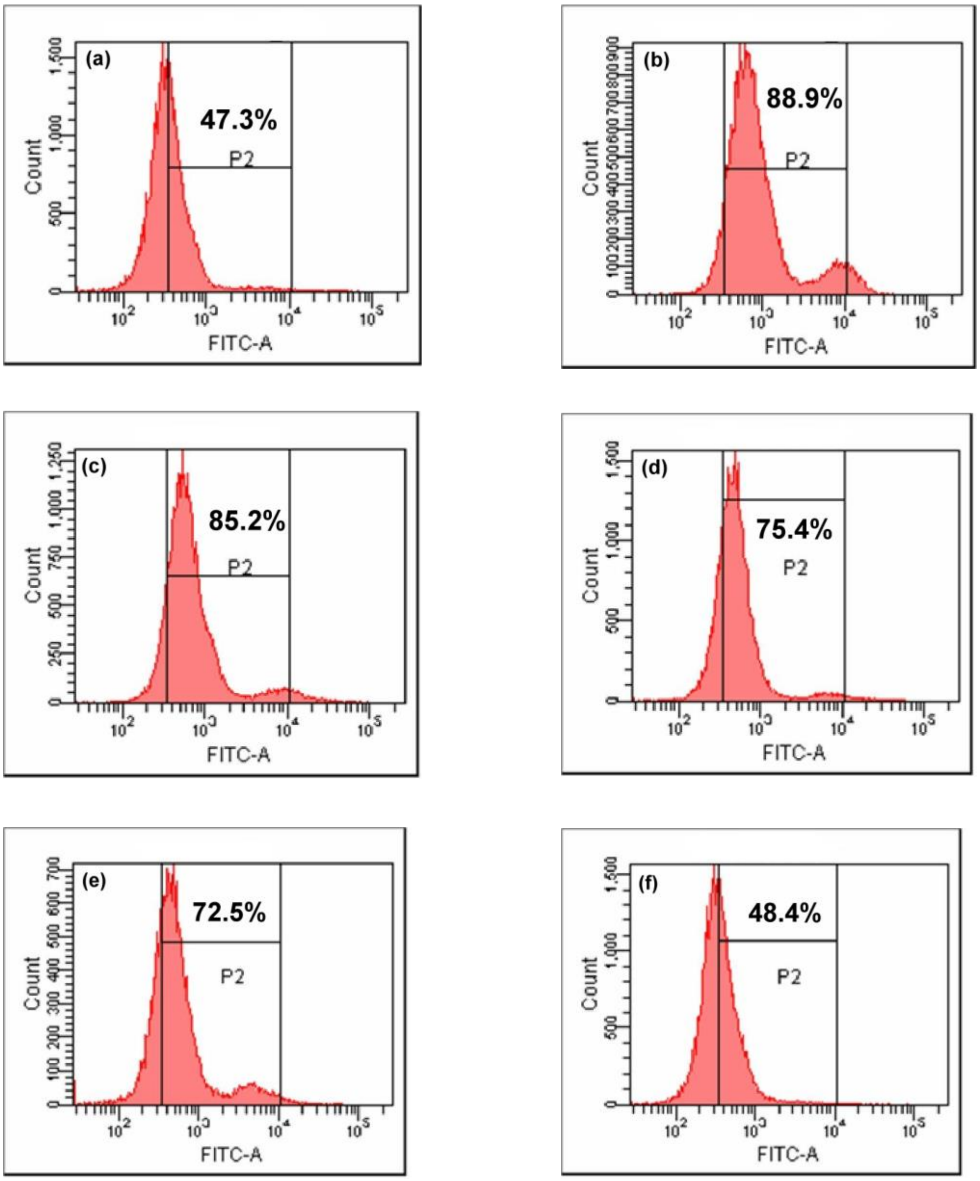
(a)
47.3%
P2
Count
FITC-A
(b)
88.9%
P2
Count
FITC-A
(c)
85.2%
P2
Count
FITC-A
(d)
75.4%
P2
Count
FITC-A
(e)
72.5%
P2
Count
FITC-A
(f)
48.4%
P2
Count
FITC-A

(B)

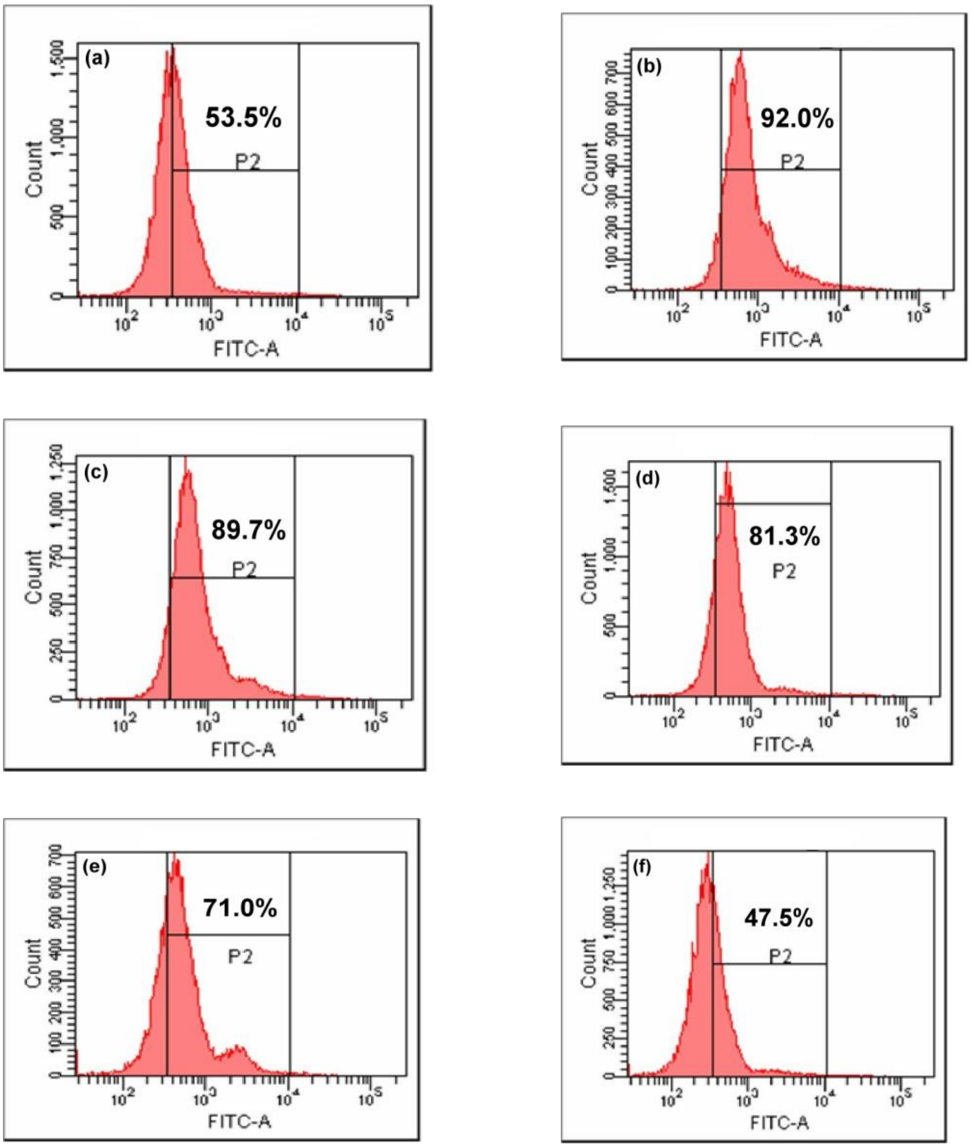
(a)
53.5%
P2
Count
FITC-A
(b)
92.0%
P2
Count
FITC-A
(c)
89.7%
P2
Count
FITC-A
(d)
81.3%
P2
Count
FITC-A
(e)
71.0%
P2
Count
FITC-A
(f)
47.5%
P2
Count
FITC-A

(C)

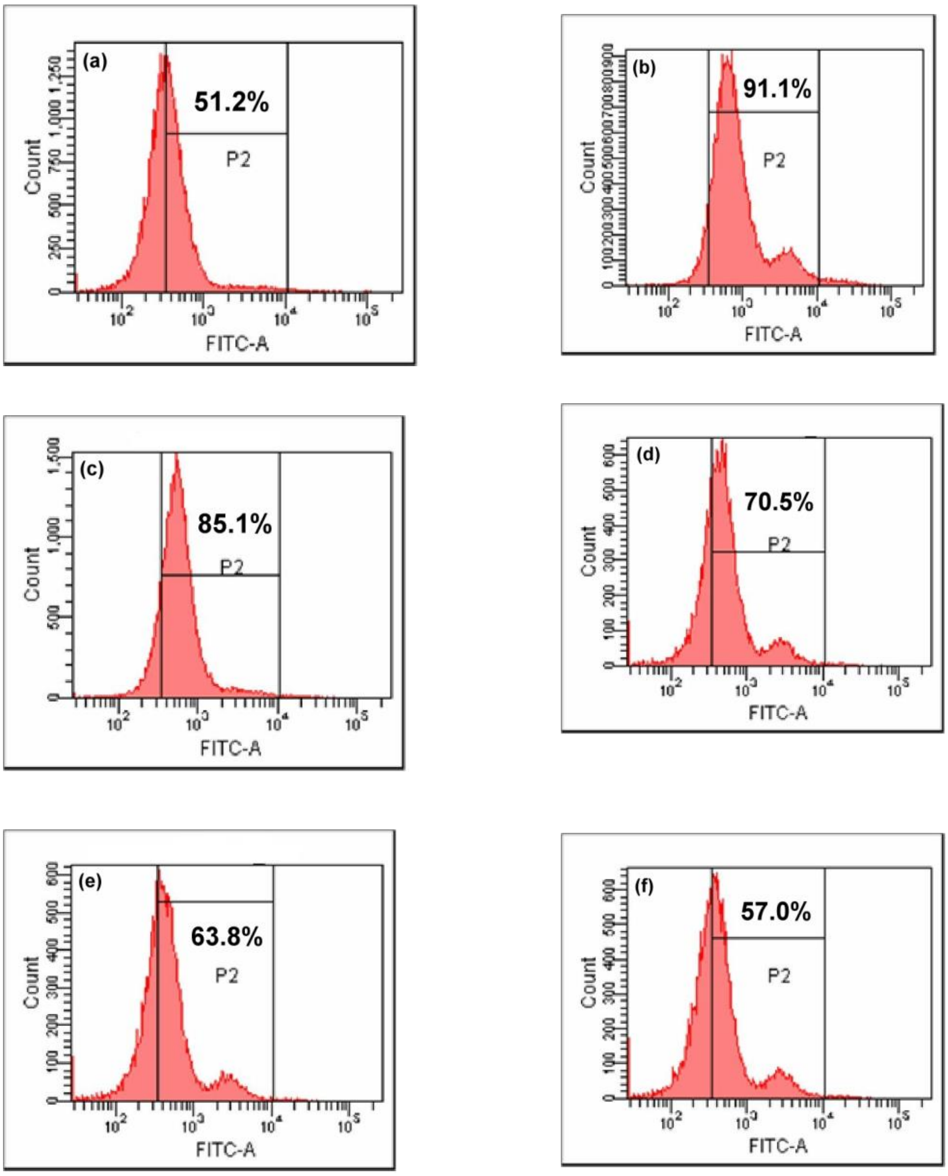

Fig. S5. The effects of ACE polysaccharides, ACE/CS and ACE/S on (A) caspase 9, (B) caspase 8 and (C) caspase 3 in Hep G2 cells for 48 h incubation.
